# Ultrasound Features of a Uterine Perivascular Epithelioid Cell Tumor (PEComa): Case Report and Literature Review

**DOI:** 10.3390/diagnostics10080553

**Published:** 2020-08-03

**Authors:** Luca Giannella, Giovanni Delli Carpini, Nina Montik, Valeria Verdecchia, Francesca Puccio, Jacopo Di Giuseppe, Dimitrios Tsiroglou, Gaia Goteri, Andrea Ciavattini

**Affiliations:** 1 Woman’s Health Sciences Department, Gynecologic Section, Polytechnic University of Marche, 60123 Ancona, Italy; luca.giannella@ospedaliriuniti.marche.it (L.G.); Giovanni.DelliCarpini@ospedaliriuniti.marche.it (G.D.C.); nina.montik@ospedaliriuniti.marche.it (N.M.); valeria.verdecchia89@gmail.com (V.V.); Jacopo.digiuseppe@ospedaliriuniti.marche.it (J.D.G.); dimitrios.tsiroglou@ospedaliriuniti.marche.it (D.T.); 2 Biomedical Sciences and Public Health Department, Anatomic Pathology Section, Polytechnic University of Marche, 60126 Ancona, Italy; gaia.goteri@ospedaliriuniti.marche.it (F.P.); g.goteri@univpm.it (G.G.); 3 Woman’s Health Sciences Department—Gynecologic Section, Polytechnic University of Marche, Via F. Corridoni 11, 60123 Ancona, Italy

**Keywords:** uterine perivascular epithelioid cell tumor, PEComa, ultrasound features, vascular pattern, imaging

## Abstract

Background: Perivascular epithelioid cell tumors (PEComas) are rare mesenchymal tumors. One of the most frequent localizations of PEComas is the female genitourinary tract, and the uterus is the most involved site after the kidney. Correct preoperative diagnosis is rarely achieved due to the presence of nonspecific imaging features. We report a case of a uterine PEComa with particular reference to ultrasound’s role in characterizing this rare occurrence. Case presentation: a 45-year-old White woman came to our observation for cyclic abdominopelvic pain and chronic constipation. The pre-surgical ultrasound examination showed a heterogeneous tumor that was 4 cm in size, localized on the right anterolateral uterine wall. The mass had well-delimited borders and a central hypoechoic portion. The use of color Doppler showed a rich, irregular vasculature in the center with low impedance. The preoperative diagnostic hypothesis was of a smooth muscle tumor of uncertain malignant potential. After careful counseling, a surgical approach was decided upon, including a total laparoscopic hysterectomy with bilateral salpingectomy. The histological and phenotypical features were consistent with a uterine PEComa. At the last follow-up, two years after surgery, the patient is alive and well. Conclusions: Uterine PEComa is a rare occurrence that should be included in the differential diagnosis of uterine wall tumors. It can appear as a small uterine mass with heterogeneous echogenicity and a rich vascular pattern during an ultrasound evaluation. This diagnostic suspicion may assist in better surgical planning.

## 1. Background

Perivascular epithelioid cell tumors (PEComas) are a group of rare tumors defined as mesenchymal tumors composed of histologically distinctive perivascular epithelioid cells. They have a perivascular distribution and express both melanocytic and smooth muscle markers [1]. Although most PEComas have a morphologically distinctive capillary architecture, further studies need to characterize these non-myometrial tumors [2].

One of the most common primary sites for PEComas is the female genitourinary tract, and the uterus is the most involved site after the kidney [3]. Patients with a uterine PEComa have a wide variety of clinical outcomes [3]. The risk of these tumors’ aggressive behavior has been linked to several factors that are evaluable using histology following surgical resection [4].

Folpe and Schoolmeester divided PEComas into three categories: benign, uncertain malignant potential (UMP), or cancerous lesions. Five morphological and pathological criteria are considered: gross size ≥5 cm, high-grade nuclear features, necrosis, vascular invasion, or a mitotic rate higher than or one per 50 HPF [1,3]. Those lesions that meet four out of five criteria described above are classified as malignant, and benign or UMP otherwise [1,3].

Usually, its correct preoperative diagnosis is rarely achieved due to the presence of nonspecific imaging features. These uterine structural lesions are often confused with fibroids, affecting proper surgical planning. Hence, better characterization of these lesions with imaging is important. We report a case of uterine PEComa with particular reference to the role of ultrasound imaging in recognizing this rare occurrence. A literature review on the topic was also performed, emphasizing the most recurrent ultrasound appearances.

## 2. Case Presentation

A 45-year-old Caucasian woman came to our attention with a diagnosis of a right intraligamentary lesion found in the ultrasound and pelvic magnetic resonance imaging. She suffered from cyclic abdominopelvic pain and chronic constipation. The patient’s past medical history included hypothyroidism and breast cancer treated with quadrantectomy, axillary dissection, and radiotherapy, followed by tamoxifen therapy for five years.

**Figure 1:**
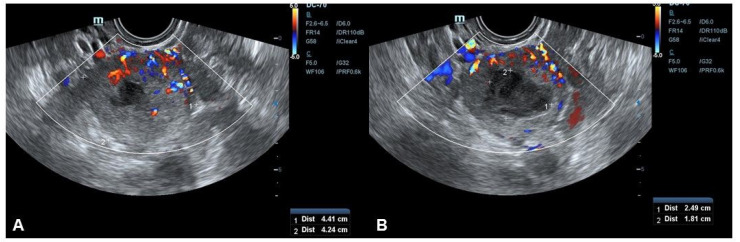
Transvaginal ultrasound showing a subserosal mass 4 cm in size with well-delimited borders and a central hypoechoic portion that was 25 × 18 mm (**A**,**B**). Color Doppler showed a tumor with a rich, irregular, central vascular network (**A**) (color score 3).

The transvaginal ultrasound examination (Mindray DC-60 Exp, Shenzhen, China) presented a heterogeneous tumor that was 44 × 42 × 37 mm in size, localized on the right anterolateral uterine wall. The mass had a subserosal location, with well-delimited borders and a central hypoechoic portion that was 25 × 18 mm (Figure 1). According to the International Ovarian Tumor Analysis (IOTA) classification system [5], color Doppler showed a tumor with a rich, irregular, central vascular network (Figure 1) with low impedance (color score 3). The preoperative diagnostic hypothesis was a smooth muscle tumor of uncertain malignant potential (STUMP). Additional exams, including tumor markers (CA-125, CEA, CA 19.9, CA 15-3), were regular.

**Figure 2:**
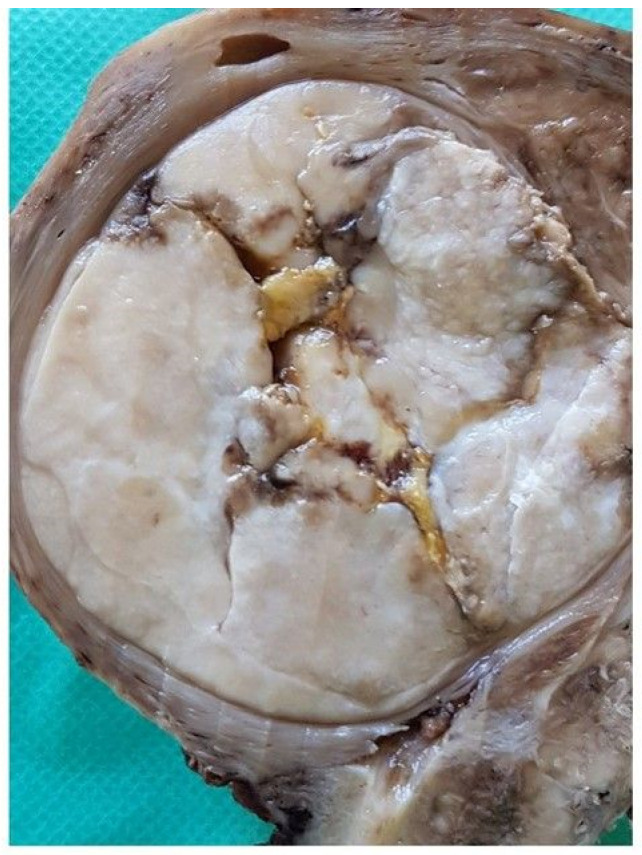
Gross appearance of the tumor after fixation in formalin. A tumor with a largest diameter of 4 cm was present beneath the serosa; the margins were well-delimited and a central area of cavitation was visible.

After careful counseling with the patient, it was decided to perform a total laparoscopic hysterectomy (TLH) with a bilateral salpingectomy. In the endoscopic view, there was no evidence of other lesions in the abdominopelvic cavity. The uterus was removed without superficial damage to avoid any possible pelvic contamination. During the laparoscopy, the uterine mass was similar to a uterine fibroid. Macroscopically, it appeared as an exophytic neoformation of the right uterine wall with regular margins and surfaces (Figure 2).

**Figure 3:**
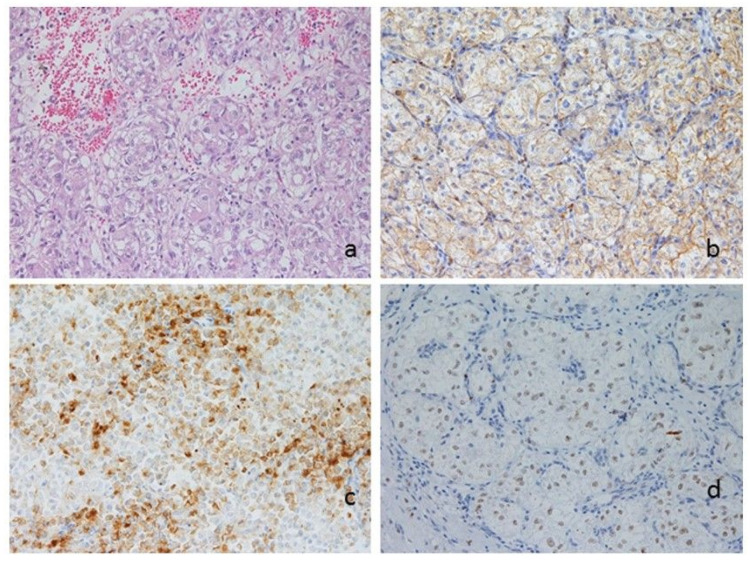
Morphologic and phenotypic features of the tumor: (**a**) nested architecture with thin-walled vascular spaces and a composition of large cells with a clear to granular eosinophilic cytoplasm, round to ovoid nuclei and prominent nucleoli (hematoxylin and eosin, 400×); (**b**–**d**) immunohistochemical stainings returned positive for cathepsin K (**b**), HMB-45 (**c**), and TFE3 (**d**, 400×).

Histologically, the tumor showed a nested architecture with thin-walled vascular spaces and was composed of large cells with a clear to granular eosinophilic cytoplasm, round to ovoid nucleus, and prominent nucleoli (Figure 3a). According to previous studies [6], to better differentiate a PEComa from smooth muscle tumors, the immunohistochemical analysis was performed. It showed cytoplasmic positivity for cathepsin K (Figure 3b) and HMB-45 (Figure 3c), nuclear positivity for TFE3 (Figure 3d), and focal positivity for SMA.

According to the prognostic systems of Folpe et al. and Schoolmeester et al. [1,3], the lesion showed microscopic characteristics of UMP, which made clinical follow-up mandatory. The monitoring was performed with a gynecological evaluation (clinical and ultrasound assessment) and chest/abdomen/pelvis CT every six months. Two years after surgery, the follow-up was negative.

## 3. Discussion

The pre-surgical diagnosis of malignant uterine lesions is often complicated, and there is no clear evidence about sonographic characteristics that can raise such suspicion. While there are study groups for the ultrasound characterization of ovarian and endometrial pathologies (IOTA and IETA groups) [7,8], we do not yet have study groups for mesenchymal tumors. Furthermore, there are no specific serum markers associated with these uterine lesions. Specifically, the diagnosis of a PEComa is given by the combination of histopathological evaluation and immunohistochemical markers [6]. PEComas are characterized by their positivity to myoid (such as desmin, SMA, muscle-specific-actin, muscle myosin, and calponin) and melanocytic markers (such as HMB-45, Melan-A/MART-1, tyrosinase, and MiTF) [6]. Cathepsin K represents an additional immunohistochemical marker that is frequently and strongly expressed in PEComas [6]. In some cases, these lesions may present TFE3 rearrangements [6]. Extrarenal TFE3-associated PEComas showed aggressive behavior during follow-up in 52% of cases [6]. In general, the prognosis is favorable [9]. However, there are cases with aggressive behavior that present distant metastases in 32% of cases, or disease recurrence after treatment in 58% of women [4]. Finally, although rarely, uterine PEComas can be found in pregnancy and may cause severe obstetric complications, such as uterine rupture, hemoperitoneum, or retained placenta [10].

Usually, the treatment of uterine structural lesions is done using a laparoscopy. Laparoscopic removal of uterine lesions through the trocars involves their “morcellation” into small pieces. In 2014, the US Food and Drug Administration reported that laparoscopic morcellation should not be performed because of the risk of disseminating unrecognized sarcomatous lesions [11,12]. To date, it is recommended to perform a laparoscopy that provides morcellation only in patients with a “low risk” of sarcomatous lesions after preoperative magnetic resonance imaging. Otherwise, you must have the option of performing protected morcellation in a bag [11].

Regardless of the latest technological progress regarding imaging, diagnosing a malignant uterine tumor is still tricky. The significant volume and fast growth in premenopausal women were once considered vital suspected signs (e.g., increasing by six weeks’ gestational size within one year) [13]. Nevertheless, a lot of clinical data contrasted this evidence [14].

**Figure 4:**
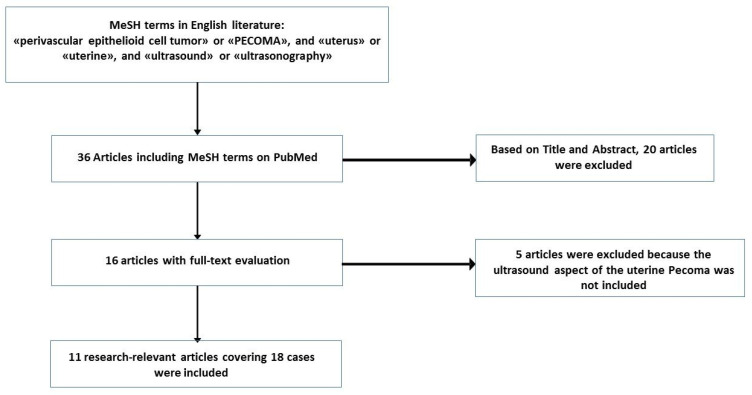
Search strategy flowchart.

To date, around 89 cases of uterine corpus PEComas have been reported in the English language literature [15,16]. However, there are no precise preoperative imaging characteristics suggestive of a PEComa. The electronic medical database PubMed was used for research, combining the following terms: (“perivascular epithelioid cell tumor” or “PEComa”) and (“uterine” or “uterus”) and (“ultrasound” or “ultrasonography”). There were eleven articles in the English literature, referring specifically to the uterine PEComa ultrasound appearance for a total of 19 cases (including the present report) [15,16,17,18,19,20,21,22,23,24,25] (Figure 4).

The mean reported diameter of PEComa was about 7 cm. In 6/19 cases, only the ultrasound dimensional features were indicated, while in the remaining 13 cases, additional ultrasound features were also reported (Table 1). Most cases revealed heterogeneous echogenicity during the ultrasound examination. In the paper from Tirumani et al., one case of uterine PEComa was described as a well-circumscribed heterogeneous mass with no cystic areas or significant vascularity during the Doppler examination, being confused with uterine fibroid [21]. Four authors described malignancy characteristics (high central vascular network with low impedance and an imprecise border of the tumor but not hypo/anechoic or cystic area) that led to confusing it with a sarcoma [15,18,19,21]. The presence of a hypo-anechoic area inside the tumor led to a diagnosis of fibroid or degenerate leiomyoma in two cases [16]. Only in one case, the appearance of a cystic area with mixed echo and rich vascularization was confused with an ovarian cyst torsion [16].

In our case, the mass’s vascularization appeared particularly accentuated (color Doppler score 3), in line with the existing literature [18,19]. Indeed, 8 cases out of 10 reported vascularization data showing significant vascular modifications characterized by a rich central vascular network (Table 1). Conversely, the size of our mass was smaller than the average reported in the literature. Our suspicion of a potential malignant mass in the preoperative ultrasound was significant for planning the surgery. Furthermore, the presence of a risk factor for uterine malignancy, such as previous tamoxifen use, played a role in the decision-making process [11]. A non-conservative approach, with a TLH and bilateral salpingectomy, was performed after considering the patient’s age and no desire for future pregnancies.

The lack of specific clinical and radiological findings makes the diagnosis and the management of PEComas challenging [9]. The ultrasound diagnosis of malignant uterine masses appears difficult. No ultrasound pattern has been described in the literature as characteristic of STUMP, leiomyosarcoma, or PEComas [26]. Ultrasound evaluation of a uterine lesion can reveal malignancy features, such as central necrosis, irregular margins, and a high color Doppler score. However, the positive predictive value of these characteristics is low, as they can also be present in benign lesions [15]. Magnetic resonance imaging can better define the lesion’s internal structure, but the reported appearances are varied [9]. There are no imaging techniques that can discriminate against these lesions from other benign or malignant gynecological tumors [9]. However, the search for one or more of these features can be useful when suspecting preoperative malignant uterine mesenchymal pathology, playing a crucial role in the treatment choice [26]. Considering the histological origin of PEComas, the vascular pattern study may have a pivotal role in the diagnostic process.

## 4. Conclusions

A uterine PEComa is a rare occurrence that should be included in the differential diagnosis of uterine wall tumors and when it appears as a small uterine mass. For the first time, the present review reported all the ultrasound appearances of an infrequent and challenging gynecological pathology. Although the ultrasonographic features were varied, some recurrent imaging characteristics, such as heterogeneous echogenicity and rich vascular patterns, may be of help in clinical practice. This diagnostic suspicion may assist in better surgical planning.

## Figures and Tables

**Table 1:** Ultrasonographic features of uterine PEComas.

Heading	No.	Dimensions (cm)	Echogenicity	Position	Vascularization	Cystic Areas	Margins	Suspected Diagnosis
Giannella, (the present report)	*n* = 1	4.7 × 3.3 × 4.5	Heterogeneous	SS	Central rich vascularization	Intense central anechoic portion	Well-delimited borders	Smooth muscle tumors of uncertain malignant potential
Liu CH [15] 2019	*n* = 1	10	-	-	Rich flow	-	-	Uterine sarcoma
Shan [16] 2018	*n* = 4	10.8 × 10.1 × 9.5	Heterogeneous	-	Rich blood supply	Cystic area with mixed echo	-	Left ovarian cyst torsion
		3.5 × 2.9 × 1.5	-	-	Cord-like blood flow	Hypoechoic area	-	Uterine leiomyoma
		5.3 × 4.4 × 3.8	-	-	Abundant dotted blood supply	Multiple areas with no echo	-	Degenerate uterine leiomyoma
		5.8 × 6.2 × 3.8	Hypoechoic	-	Abundant blood supply	-	-	Degenerate uterine leiomyoma
Su Kwon [17] 2017	*n* = 1	4.6	Heterogeneous	-	-	-	-	-
Socolov [18] 2016	*n* = 1	5.2 × 5.7	Hyperechogenic Heterogeneous	-	Central vascular network with low impedance	No cystic areas	Imprecise borders	Uterine sarcoma
Verbeeck [19] 2016	*n* = 1	10	Granulomatous ovoid uterine tumor	-	Rich vascular network with low impedance	-	-	Uterine sarcoma
Fitzpatrick [20] 2016	*n* = 1	10.5 × 9.0 × 12.0	-	-	-	-	-	-
Tirumani [21] 2014	*n* = 2	-	Heterogeneous	-	No significant vascularity in Doppler profile	No cystic area	Well-defined margins	Uterine leiomyoma
Tirumani [21] 2014	*n* = 1	14	Heterogeneous	-	-	-	Poor delimitation of margins	Uterine sarcoma
Yu [22] 2014	*n* = 1	4.5 × 4.5 × 3.5	-	SS	-	-	-	-
Issat [23] 2012	*n* = 1	9.2 × 7.6	Hyperechogenic Partially solid mass	-	-	Partially cystic	-	-
Liu JL [24] 2009	*n* = 1	8.1 × 7.2 × 6.4	-	-	-	-	-	Uterine leiomyoma
Gan [25] 2007	*n* = 3	3.5 × 2.5 × 2.0	-	SS SS	-	-	-	Uterine leiomyoma
		8.5 × 4.3 × 6.4						
		5.5 × 5.0 × 4.5						

SS: subserosal.
